# Editorial: Masculinities, empathy, care, and non-violence

**DOI:** 10.3389/fsoc.2026.1835209

**Published:** 2026-04-08

**Authors:** Tatiana Moura, Haydée Caruso, Júlia Garraio

**Affiliations:** 1 Universidade de Coimbra Centro de Estudos Sociais, Coimbra, Portugal; 2 Department of Sociology, University of Brasilia, Brasília, Brazil

**Keywords:** care, gender, juvenile detention center, masculinities, non-violence

As gender studies increasingly move from critique toward transformative practice, the Research Topic *Masculinities, empathy, care, and non-violence* responds to an urgent contemporary challenge. It builds on the recognition that boys and men play a central role in the reproduction, prevention, and interruption of violence across generations. Rather than focusing solely on deconstructing hegemonic masculinity, this Research Topic advances a proactive approach in which empathy, care, and non-violent engagement are understood as foundational elements in redefining masculinities.

The issue places youth at the center of analysis, recognizing adolescence as a critical stage in which gender roles, emotional repertoires, and social expectations are consolidated. Across diverse empirical contexts, the articles explore masculinities in relation to violence, care practices, creative expression, media representations, persistent gender norms, and global crises such as the COVID-19 pandemic. Together, they call for innovative theoretical, methodological, and political responses to the enduring association between masculinity and violence.

## Why this Research Topic matters

This Research Topic brings together seven articles based on research conducted in Portugal, Spain, and Croatia—contexts historically shaped by rigid gender norms and a strong division between public (masculine) and private (feminine) spheres. Despite decades of progressive legal and policy reforms, traditional models of masculinity and sexist cultural patterns remain deeply embedded in everyday life. These contexts therefore provide fertile ground for examining how care, empathy, and non-violence may disrupt norms that equate masculinity with domination, emotional restraint, and the use of force.

This Research Topic contributes to sociological and feminist debates by advancing several conceptual and analytical shifts. First, it moves from critique to care, shifting attention from the exclusive deconstruction of violent masculinities to the active construction of compassionate and caring alternatives grounded in feminist theory and practice. Second, it foregrounds youth as central agents, emphasizing both the vulnerability and transformative potential of adolescents, particularly those exposed to violence, institutionalization, or social marginalization. Third, it adopts an intersectional and situated perspective, demonstrating how masculinities are shaped by place, social inequalities, migration, race, sexuality, and institutional contexts. Fourth, it highlights methodological innovation, privileging ethnography, participatory methods, and creative approaches that engage young people as subjects rather than objects of research. Finally, it responds to global crises, such as the COVID-19 pandemic, showing how moments of disruption make care practices—and their absence—particularly visible in gendered experiences.

Taken together, the articles urge readers to rethink masculinities through the lenses of empathy, care, and non-violence, while calling for sustained research and practical interventions rooted in young people's everyday lives.

## Contributions that educate and transform

The articles assembled in this issue offer original perspectives on how masculinities are learned, embodied, negotiated, and contested in contexts marked by violence and inequality. They also generate insights with clear implications for education, social intervention, and public policy.

Moura's article, “*Transforming harmful representations, investigating violence and empowering care practices in masculinities*” advances a feminist theory of caring masculinities. Drawing on empirical research conducted in Portugal, Moura examines how patriarchal masculinities are reproduced through emerging forms of violence among boys. She argues that care, empathy, emotional expression, and cooperation are key resources for countering violent masculinities. The article also highlights the importance of engaging men and boys in feminist debates and activism, particularly through educational programs and the early promotion of fathers as caregivers.

Several articles focus on young people in custodial or semi-custodial settings, revealing how institutional practices shape masculine identities. Mascarenhas, in “*Straight from foster care to the youth detention center? The (mis)paths of child protection and juvenile justice policies in the construction of violent masculinities*,” examines young men in Portuguese youth detention centers by analyzing public policies, life trajectories, and professional practices. She shows how child protection and juvenile justice systems often contribute to the construction of violent masculinities by prioritizing control, punishment, and fear over care and dignity. Mascarenhas argues that meaningful change requires trauma-informed and gender-aware professional training.

In “*I stayed on the street, as if there was no COVID-19”: enacting practices of care with youth at risk of violence and social exclusion in Portugal*, *Ferreira* addresses the impact of the COVID-19 pandemic on young people in Portuguese detention centers, demonstrating how public health measures intensified isolation, vulnerability, and emotional deprivation. Her analysis underscores the centrality of care practices and support networks in sustaining non-violent futures, particularly in times of crisis.

Caruso's article, “*When fiction meets reality: ethnofiction as a way of constructing youth narratives about delinquent paths*,” examines ethnofiction as a methodological tool for accessing young people's experiences in juvenile detention centers. Based on a 2022 study in Portugal, the research invited participants to construct narratives through a fictional character. These stories illuminated dimensions of their lives that are often difficult to articulate, including childhood, schooling, fractured family ties, community relations, encounters with the police, institutionalization, and uncertain futures. Caruso argues that ethnofiction functions as a powerful reflexive and ethical resource, enabling young people to narrate their social worlds.

Migration and border regimes are central to the article by Alonso et al. “*Embodying two shores of the Mediterranean Sea: the liminal masculinity of minors migrating alone to Spain.”* Focusing on unaccompanied migrant minors in Spain, the authors adopt an intersectional perspective to analyze the metaphorical borders—legal, racial, generational, and gendered—that shape these young men's lives. They propose the concept of liminal masculinities to capture how migrant youth negotiate masculinity in transitional spaces marked by vulnerability, violence, and resilience.

In the Croatian context, Matković et al. examine gender norms in juvenile correctional institutions: “*Challenging the status quo: gender norms in Croatian juvenile correctional settings*.” Their findings reveal the persistence of rigid, hierarchical gender norms, particularly among boys, reinforced by the institutional organization of correctional settings. The authors argue that effective violence prevention requires gender-sensitive programs targeting both young people and professionals, with particular attention to discriminatory attitudes toward sexual minorities and Roma youth.

The final article shifts the focus to media representations. In “*Caring masculinities and the politics of feminist representation in the Portuguese media*,” Santos et al. analyze Portuguese television advertisements that portray so-called “caring masculinities.” Although these representations appear to challenge hegemonic norms by foregrounding fatherhood and caregiving, the authors demonstrate that they largely reproduce conventional masculine expectations.

The articles in this Research Topic contribute to feminist and sociological debates on masculinities by centering care, empathy, and non-violence as analytical and political priorities. They also highlight the need for comparative research that bridges the Global North and the Global South, recognizing shared challenges as well as contextual specificities.

